# To give or not to give, and to whom?—These are the questions! Determinants of selective organ donation intentions

**DOI:** 10.3389/fpsyg.2025.1625981

**Published:** 2025-10-22

**Authors:** Alexandra Cobzeanu, Cristian Opariuc-Dan, Ioan-Alex Merlici, Bogdan Mihail Cobzeanu, Ionut Tanase

**Affiliations:** ^1^Department of Education Sciences, Faculty of Psychology and Education Sciences, Alexandru Ioan Cuza University of Iasi, Iasi, Romania; ^2^Faculty of Law and Administrative Sciences, Ovidius University, Constanta, Romania; ^3^Department of Biomedical Sciences, Faculty of Medical Bioengineering, “Grigore T. Popa” University of Medicine and Pharmacy, Iasi, Romania; ^4^Department of Stomatology II, Faculty of Dentistry, Carol Davila University of Medicine and Pharmacy, Bucharest, Romania

**Keywords:** posthumous organ donation, utilitarianism, empathy, moral norms, subjective norms

## Abstract

**Introduction:**

This study examined the psychological factors influencing intentions for Posthumous Organ Donation (POD). Specifically, we focused on beliefs associated with POD, perceived subjective and moral norms derived from the extended Theory of Planned Behavior, empathic concern, and a facet of utilitarianism, namely instrumental harm. We explored these variables in relation to participants' overall intention to donate organs, as well as their intention to donate in specific contexts: to a child and to an adult with disabilities.

**Methods:**

A total of 1,896 Romanian adults aged 18 to 80 participated in the study. Data were analyzed using a moderated structural equation modeling approach to assess the role of medical mistrust, disgust-related factors (Ick and Jynx), bodily integrity concerns, perceived benefits, norms, empathic concern, and instrumental harm in predicting POD intentions.

**Results:**

Medical mistrust, Ick, Jynx, and bodily integrity concerns had significant negative effects on POD intentions. In contrast, perceived benefits, as well as subjective and moral norms, positively predicted POD. Empathic concern did not significantly moderate POD intentions. However, instrumental harm was negatively associated with all forms of POD (general, to a child, and to an adult with disabilities). Participants reported the highest POD intentions when the recipient was a child with disabilities, and the lowest intentions for general POD.

**Discussion:**

The findings suggest that while normative beliefs and perceived benefits enhance willingness to engage in POD, concerns about bodily integrity, mistrust, and aversion responses hinder such intentions. The role of instrumental harm underscores the ethical complexities in organ donation decision-making. Context also matters, as donation to vulnerable recipients (e.g., children with disabilities) elicited the strongest support. These results highlight the importance of addressing mistrust and disgust-related barriers, while strengthening moral and normative frameworks, to promote organ donation.

## Introduction

Organ transplantation saves thousands of lives and is one of the most widely used and cost-effective solutions for patients dealing with organ failure. However, the demand for organs significantly exceeds the supply (López et al., 2018). While many individuals are willing to donate their organs posthumously to help those in need, the actual number of donations remains low due to various factors, including the refusal of donors' families to give consent and the poor physical health of some potential donors (Ferguson et al., 2019). Recent data also highlight that, contrary to the general European trend and the significant improvements in Romania's transplantation system, the number of donors in Romania has decreased significantly in recent years (Bacuşc et al., 2022), with a donation rate of 3.44 donors per million residents (Bacuşc et al., 2023). The legal framework for organ and tissue donation in Romania is established by Law 95/2006. Romania and six other European Union member states have chosen to participate in an opt-in (i.e., informed or voluntary) consent system (Scholz, 2020). The Romanian National Transplant Agency oversees organ donation and transplant operations in Romania, managing six regional centers and 33 affiliated units (Cotru et al., 2020).

Several factors contribute to individuals' POD intentions, including (but not limited to) their religious and spiritual beliefs, cultural group, family members' influence, and socioeconomic status (Mekkodathil et al., 2020). Moreover, individuals with high POD intentions exhibit various motivations, including financial incentives (i.e., relatives receiving financial compensation for their donation), social or religious obligations, and a desire to save the lives of others (Guedj et al., 2011). Furthermore, POD intentions might also be influenced by various situational factors, including the relationship with the patient who would receive the organs and the specific organs that will be donated (Skowronski, 1997). Other potentially relevant factors include knowledge related to organ donation, a general tendency to engage in prosocial behaviors, and a positive attitude toward other procedures that involve medical interventions on deceased bodies (e.g., autopsies; Febrero et al., 2019).

Ethical values and morality-related variables might also play a relevant role. For instance, utilitarians might see more benefits in organ donations, approving transplants as a beneficial solution that would help a higher number of individuals. Conversely, deontologists might be more concerned about the wellbeing and dignity of deceased donors, thereby being more reluctant about organ donation (Chukwuneke and Ezenwugo, 2022). In the current study, we aimed to investigate some of these factors to provide valuable information for developing targeted interventions to increase organ donation rates, given the documented number of lives saved through this procedure (Girlanda, 2016) but also to decrease organ trafficking, which is a direct consequence of the disparity between organ availability and patients awaiting organ transplantation (Da Silva and Frontera, 2015).

Furthermore, previous studies conducted in Romania suggested that, generally, the low performance of the Romanian transplant system can be attributed to several reasons, including insufficient funding, a shortage of specialized medical personnel, and an uneven allocation of resources among various medical facilities, in addition to poor medical education, the lack of organ and tissue banks, the requirement for family consent, and inadequate communication between different parts of the system (Grigoras et al., 2010; Holman and Beatrice, 2016). A 2010 Eurobarometer indicated that 40% of Romanians were against organ donation; more than half of them could not explain why, 15% reported medical mistrust, and 17% offered religious reasons for their refusal (Samoila, 2010). Furthermore, Cotru et al. (2023) reported that the most significant positive predictor of Romanians' willingness to donate was the belief that one is assisting others by donating their organs following brain death. Conversely, the most negative predictor suggested by the authors was related to bodily integrity. Similar findings were reported by (Holman et al. 2013), who reported that knowledge and concerns regarding posthumous manipulation of the body were among the most important predictors.

However, the majority of these studies focused on consent and awareness for organ donation (Cotru et al., 2023; Grigoras et al., 2010), media and social representations (Holman and Karner-Huţuleac, 2017; Petre and Bban, 2022; Todeanc et al., 2019), and the malfunctions of the Romanian national transplantation system (Holman and Beatrice, 2016). Additionally, the majority of these studies highlighted the pressing need to raise awareness about organ donation (Bacuşc et al., 2022). For instance, educational initiatives in schools and colleges are recommended by the Council of Europe (Holman and Beatrice, 2016) and can play a crucial role in this regard. However, these efforts require further empirical evidence to inform the development of effective, targeted strategies, and the present study aims to contribute to this goal.

### Beliefs associated with organ donation

This study focused on five sets of beliefs related to organ donation, i.e., medical mistrust, bodily integrity, the Ick factor, the jinx factor, and perceived benefits (Morgan et al., 2008b). *Medical mistrust* represents an overall reduced willingness to trust medical personnel, often as a consequence of negative experiences related to medical needs (e.g., marginalization, neglect, or medical errors; Ghoshal et al., 2022). An individual who has a background of distrust in the medical field may approach the process of deciding whether to donate organs with a skeptical perspective, affecting their willingness to take part in and support organ donation initiatives. However, the current research literature offers mixed findings on the role of medical mistrust in this regard. The majority of the studies suggest that medical mistrust has a negative impact on POD intentions (Williamson et al., 2019; Ghoshal et al., 2022). However, some previous studies suggest that medical mistrust might have a limited or insignificant effect on POD intentions (Russell et al., 2012). Thus, future research, such as the present study, is needed to better understand the role of medical mistrust, address people's trust issues, and tailor communication strategies that acknowledge and alleviate concerns related to medical mistrust, particularly in the context of organ donation.

*Bodily integrity* refers to concerns related to preserving the integrity of deceased individuals' bodies in order to maintain their dignity (Ruggieri et al., 2023). This factor represents a significant barrier that usually decreases individuals' POD (Viens, 2016). Previous studies consistently suggest that participants with high levels of bodily integrity beliefs are more likely to refuse to donate their organs (O'Carroll et al., 2011). Moreover, bodily integrity might have a negative effect not only on POD intentions but also on general attitudes related to organ donation (Arisal and Atalar, 2020). Individuals who are concerned with bodily integrity might perceive organ transplants as dehumanizing and as a violation of dignity and privacy, thus being more likely to reject both donating and receiving organs (Pfaller et al., 2018).

*The Ick factor* represents a strong reaction of disgust and repulsion to the idea of organ donation (O'Carroll et al., 2011). Compared to medical mistrust or perceived benefits, which imply a stronger focus on the cognitive aspects related to organ donation, the Ick factor is strongly influenced by emotions, with disgust playing a particularly significant role in determining individuals' rejection of organ donation (Khoshravesh et al., 2019). Individuals with high levels of the Ick factor might be easily disgusted even by terminologies or descriptions that present organ transplants as morbid or gory, thus making them less willing to approve organ donation (Bramstedt, 2022). Previous studies suggest that non-donors exhibit higher levels of the Ick factor than those with high POD intentions (O'Carroll et al., 2011). These results are also supported by more recent findings, which suggest that individuals who refuse to donate their organs or are undecided present higher levels of the Ick factor compared to those who are willing to donate (Miller et al., 2019).

*The Jinx factor* represents a set of unwarranted fears and superstitions related to the potential negative effects of organ donation (Andrews et al., 2016). This factor was previously associated with lower POD intentions and with more negative attitudes toward organ donation (QuIck et al., 2016). Moreover, individuals who refuse to donate their organs and those who are undecided on their stance on organ donation usually present significantly higher levels of the Jinx factor (Miller et al., 2019). Overall, the Ick and the Jinx factors, both representing negative affective attitudes related to organ donation, appear to correlate and predict lower POD intentions (Clark et al., 2023).

Finally, *the perceived benefits* comprise the beliefs related to the perceived positive and significant impact of becoming an organ donor, which helps other individuals survive (Morgan et al., 2008b). These beliefs represent relevant predictors of positive attitudes toward organ donation (Alsalem et al., 2020). More specifically, individuals who perceive more benefits from donating organs are more likely to become donors or to display positive attitudes toward organ donation. Moreover, this effect appears to be stronger for perceived personal benefits compared to perceived benefits for other individuals (Cohen and Hoffner, 2013).

### Perceived subjective and moral norms

POD intentions are also influenced by several factors that can be examined through the lens of the Theory of Planned Behavior (TPB; Ajzen, 1991). According to this theoretical approach, an individual's willingness to perform a behavior is determined by their attitudes, social normative perceptions, and perceived personal control over performing the behavior (Tesema et al., 2023). In line with this framework, POD intentions are shaped by personal attitudes related to organ donation (which we expanded upon in the previous section), *subjective norms* (i.e., the stances that other persons who are relevant to the individual adopt on organ donation), and perceived behavioral control (i.e., individuals' perceptions related to their ability to donate organs, despite potential obstacles; Ferguson et al., 2019). The TPB model was found to be one of the most effective models in predicting donation intentions and also allows the integration of additional predictors into the model, provided that they enhance the model's predictive power (Hyde and White, 2009).

The extended TPB perspective proposed by Hyde and White (2009) regarding organ donation encompasses perceived *moral norms*, i.e., perceptions related to the moral correctness of donation intentions. The present study explored perceived subjective (i.e., normative influences) and moral norms in predicting organ donation intentions. Studies investigating the TPB model, which includes moral norms as a predictor, have previously shown an increase (i.e., from 4% to 10%) in the model's predictive capacity for various intentions (Manstead, 2000). However, given the limited number of studies examining organ donation intentions in relation to this extended TPB model, the present study focused on two key components of the TPB: perceived subjective and moral norms.

### The role of empathic concern

Empathy plays a crucial role in the development of prosocial behavior, enabling individuals to share experiences, needs, points of view, and desires (Riess, 2017). Previous research consistently suggests that individuals who are empathetic are more likely to donate to those in need or engage in other prosocial behaviors (Lay et al., 2020). As we can intuitively expect, empathy appears to play a similar role in determining whether individuals approve organ donation for those in need (Wilczek-Rużyczka et al., 2014). Previous studies suggest that higher levels of empathy are associated with more positive attitudes toward organ donation. However, the relationship between empathy and POD intentions still requires further exploration. For example, previous experimental studies suggest that empathetic individuals may experience higher levels of stress when exposed to extremely vulnerable individuals, which may make them less likely to make charitable donations to those individuals (Wei et al., 2021). Thus, further research may help us better integrate the specifics of POD intentions into the broader literature examining the role of empathy in this context.

Finally, empathy might also moderate the effect of various factors on POD intentions. For instance, the effect of perceived other benefits (i.e., perceptions related to how other individuals might benefit from organ donation) was suggested to predict willingness to donate organs at high levels of empathic concern (Cohen and Hoffner, 2013). Similarly, Wilczek-Rużyczka et al. (2014) reported that participants who were willing to donate showed higher empathy than those who were unwilling to donate. Empathic individuals are more oriented toward the needs of others and, thus, might be more likely to agree to donate. However, while empathy may moderate the relationship between perceived benefits and willingness to donate, the current research literature does not provide relevant data on empathy as a potential moderator of the relationship between beliefs related to organ donation, perceived moral and subjective norms, and POD intentions. More specifically, we do not know how empathy might influence willingness to donate when considering personal risks and benefits (Cohen and Hoffner, 2013).

### How does utilitarianism influence organ donation?

Utilitarianism is generally viewed as a society-centered ethical approach, arguing that sacrificing individuals is justifiable if a larger group benefits from this decision. Conversely, the deontological approach is more centered on the patient, aiming to make decisions that avoid causing harm to the individual (Chukwuneke and Ezenwugo, 2022). To the best of our knowledge, the current literature offers limited data on the role of utilitarian tendencies (especially on the instrumental harm dimension) as predictors or moderators of POD intentions. However, it would appear that donors tend to adopt a utilitarian view on organ donation, perceiving organs as worthless after the death of the patient, while individuals who disapprove of posthumous organ donation appear to follow a deontological approach, considering that they should not make decisions for individuals who are unable to decide for themselves (de Groot et al., 2015). Moreover, when making judgments related to how donated organs should be allocated, individuals appear to be driven by utilitarian and egalitarian principles (i.e., making decisions that benefit most individuals and allowing everyone an equal chance to become a receiver of donated organs; Georgiadou et al., 2015).

Furthermore, utilitarians are more likely to perceive donations or individual sacrifices for the benefit of the group as the favorable solution to ethical dilemmas. While we can intuitively presume that utilitarians might be more likely to support organ donation, this topic still needs further research (Kahane et al., 2018). Additionally, the majority of studies on utilitarian and deontological beliefs focus on various scenarios that present ethical dilemmas, and these studies offer limited information on the decisions that utilitarians might make when they are the ones who must make a sacrifice for the benefit of the group (Kahane, 2015). Through this current study, we aimed to address the gap in research by investigating how a specific utilitarian dimension—i.e., instrumental harm—might influence individuals' willingness to donate their organs. *Instrumental harm* is a core concept of consequentialist ethical theories (i.e., ethical theories, including utilitarianism, that judge the righteousness of an action based on its results), referring to a preference for decisions that involve sacrificing a small number of individuals to benefit a larger group in dilemmas where preserving the wellbeing of all individuals is impossible (Everett et al., 2018).

According to the utilitarian approach, when confronted with ethical dilemmas, individuals ought to be treated as means to achieve the desired goal (i.e., the greatest sum of benefits for the entire group; Kahane et al., 2018). Previous studies have suggested that individuals with higher instrumental harm tendencies may be more willing to make decisions that involve self-sacrifice or the sacrifice of others in the context of moral dilemmas (Shin et al., 2022). Therefore, in the context of POD, individuals with high instrumental harm might be more willing to donate and agree with the posthumous organ donation of their organs. However, to the best of our knowledge, no previous studies have tested this claim, and the present research aims to investigate it.

### Selective organ donation: the case of children and adults with disabilities

POD intentions might be strongly determined by ensuring that the donation will have beneficial effects. Individuals might be less likely to approve organ donation when they perceive the medical system to be incompetent or when they are concerned that the organs will be received by patients they deem undeserving (Morgan et al., 2008a). Even though most individuals cannot impose selective organ donations (i.e., deciding which patients should benefit from posthumous organ donation), donors might be more willing to donate to particular groups (e.g., children and first-time recipients) compared to others (Murphy and Veatch, 2006).

For instance, previous studies have also suggested that individuals' POD intentions might be higher when they perceive the potential recipients as similar to them (Kong and Lee, 2021), favoring in-group members (over out-group members) when making allocation judgments related to POD intentions (Georgiadou et al., 2015). Children with intellectual and developmental disabilities have often been excluded as potential recipients of organ transplants, and medical authorities often focus on allocating donated organs to those who can benefit the most from them (Statter et al., 2020). However, to the best of our knowledge, no previous studies have investigated individuals' POD intentions when this involves individuals with disabilities, and the present study aimed to explore this research direction.

### Demographics and the role of religion

The current research literature offers mixed findings on the role of demographic variables in predicting individuals' willingness to donate organs. For example, Tumin et al. (2016) reported no significant effect of gender or household income on POD intentions. Conversely, Thornton et al. (2006) reported that female participants were more willing to donate their organs compared to their male counterparts, even when accounting for other covariates such as religion and age. Similar findings were also reported by Wilczek-Rużyczka et al. (2014). Moreover, the relationship between willingness to donate organs and willingness to sign donor cards appears to be moderated by religiosity, with the effect being stronger for religious individuals (Umair et al., 2023).

Overall, it appears that women, atheists, and individuals belonging to the predominant ethnic group of their respective nation may be more likely to exhibit POD intentions (Mocan and Tekin, 2007). However, it is important to note that several confounding variables might influence these tendencies. For example, individuals with lower levels of income and those belonging to ethnic minorities might be less willing to donate due to limited knowledge about organ donation, distrust of the healthcare system, or cultural habits related to death and preserving the dignity of the deceased (Li et al., 2019). Moreover, religious individuals appear to be particularly concerned about the moral implications of obtaining organs from non-deceased patients or patients who did not give their consent for donation (Doerry et al., 2022).

### The present study

This study investigated the multifaceted determinants of POD intentions and their complex interplay. We explored the roles of beliefs associated with organ donation, perceived subjective and moral norms, empathic concern, and instrumental harm in participants' overall intention to donate organs (with no other specifications about the recipient, donating to a child, or an adult with disabilities).

Furthermore, to the best of our knowledge, no previous research has explored the factors underlying organ donation intentions using the proposed factor from the extended TPB framework (i.e., perceived moral norms), in addition to perceived subjective norms (TPB), beliefs associated with organ donation, empathic concern, and instrumental harm in Romania. Moreover, these factors are explored through the lens of both overall intentions and specific donation intentions (which we refer to as selective organ donation or directed donations) to better understand the role of such parameters when discussing POD intentions.

**Figure 1:**
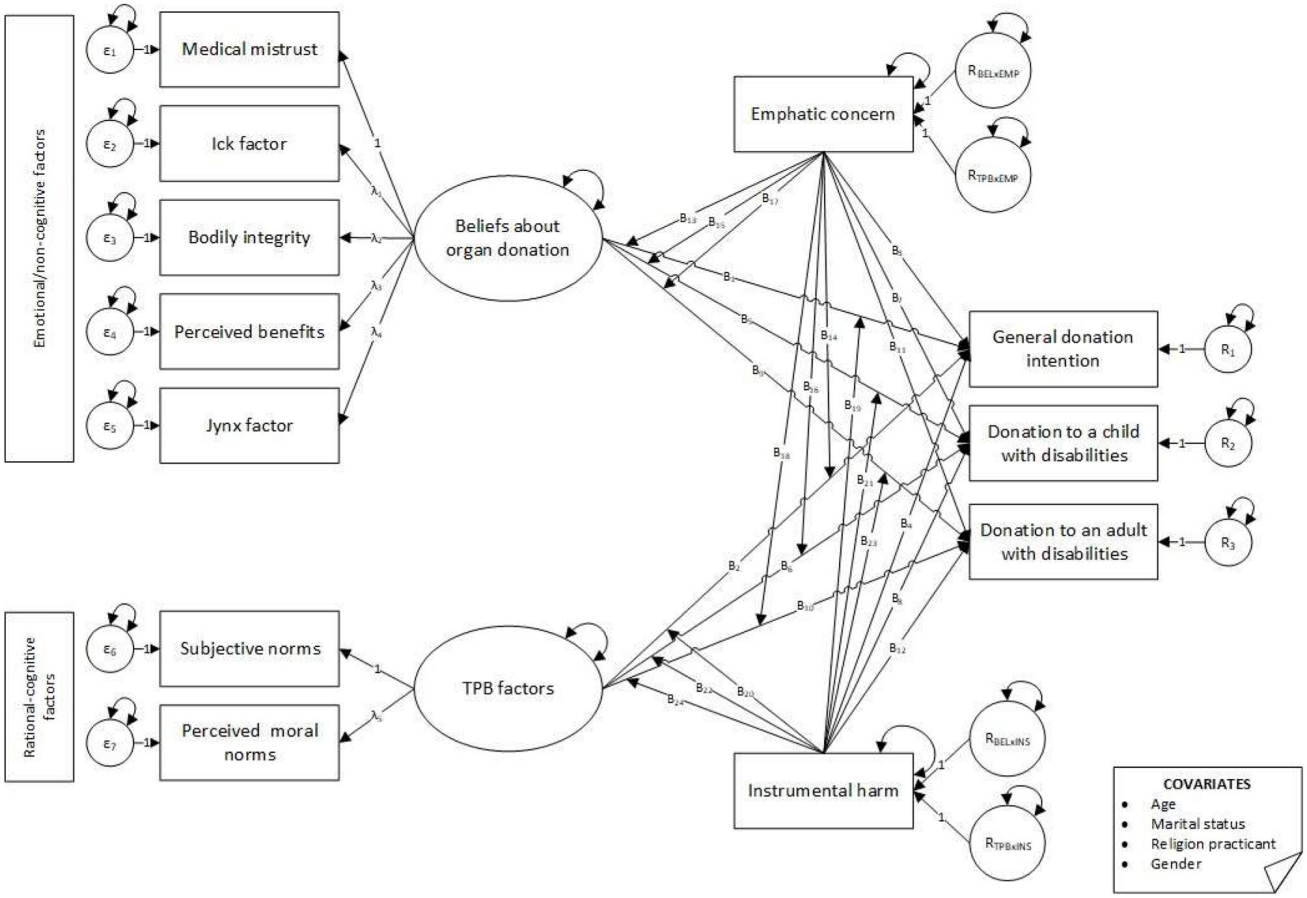
Theoretical model. Effects on donation intentions moderated by empathic concern and instrumental harm.

Based on the existing literature, the proposed research model (see Figure 1) was developed to address the following research questions: *Q1*. How do beliefs associated with organ donation, subjective norms, and perceived moral norms influence adults' POD? *Q2*. Do empathic concern and utilitarian tendencies (i.e., instrumental harm) moderate the link between beliefs about organ donation, perceived subjective moral norms, and POD? *Q3*. Are participants' POD different when they are informed about the specific characteristics of potential recipients (i.e., potential differences between overall POD, donating to a child with disabilities, or an adult with disabilities)?

To answer these questions, we formulated five hypotheses. Previous research suggested the various roles that beliefs about organ donation might play in shaping POD intentions. Medical mistrust (Williamson et al., 2019), bodily integrity (Pfaller et al., 2018), the Ick Factor (Miller et al., 2019), and the Jinx Factor (Clark et al., 2023) were previously suggested to predict lower POD intentions, while perceived benefits were suggested to predict higher POD intentions (Cohen and Hoffner, 2013). Therefore, we hypothesized that:

*H1. Beliefs about organ donations would be significantly associated with POD*.

H1.a. Medical mistrust would be negatively associated with POD; H1.b. The Ick factor (i.e., disgust) would be negatively associated with POD intentions; H1.c. Bodily integrity would be negatively associated with POD; H1.d. The perceived benefits would be positively associated with POD; H1.e. The Jynx factor (i.e., superstitions) would be negatively associated with POD intentions.

Furthermore, previous research has highlighted the utility of TPB-related factors in explaining individuals' POD, suggesting that perceived subjective and moral norms have a positive effect on POD (Hyde and White, 2009; Manstead, 2000). Therefore, we hypothesize the following:

*H2. Subjective norms and perceived moral norms would be significantly associated with POD*.

H2.a. Subjective norms would be positively associated with POD intentions;
H2.b. Perceived moral norms would be positively associated with POD intentions.

Moreover, previous research suggested the roles of empathy (George et al., 2022) and instrumental harm (Shin et al., 2022) in predicting POD intentions. A limited number of studies also suggested a potential moderating role in the relationship between beliefs about organ donation and POD (Cohen and Hoffner, 2013). Therefore, we hypothesized that:

*H3. Empathic concern and instrumental harm would be significantly associated with POD*.

H3.a. Empathic concern would be positively associated with *POD* intentions.
H3.b. Instrumental harm would be positively associated with *POD* intentions.

*H4. Empathic concern and instrumental harm would moderate the link between beliefs about organ donation, attitudes toward organ donation, and POD* intentions.

Next, while the current research literature offers limited data on individuals' POD intentions toward patients with disabilities, previous studies suggested that potential donors might prefer to donate their organs to children (Murphy and Veatch, 2006). In the current study, we aimed to address this gap in research and hypothesized the following:

*H5. POD would significantly differ depending on the recipient's general POD—i.e., no specifiers, POD to a child with disabilities, or POD to an adult with disabilities—with the lowest rate being reported when the recipient is an adult with a disability*.

Finally, we aimed to test these hypotheses while accounting for several covariates identified in previous studies as potentially significant in relation to POD intentions, namely age, gender, religion and religious practices, education, relationship/marital status, and number of children (Krupic et al., 2019; Ge et al., 2013; Randhawa and Neuberger, 2016; Shah et al., 2018).

## Materials and methods

### Participants and procedure

The initial sample consisted of 2,136 participants. However, the final sample comprised 1,896 Romanian adults aged 18–80 years (*M* = 35.71, *SD* = 12.49), with 51.32% being women. Two hundred forty participants were excluded because they did not meet the inclusion criteria. Most of the participants in the final sample were married (50.90%), unmarried (37.18%), divorced (5.38%), in a romantic relationship (5.12%), or widowed (1.37%). University degrees (62.92%) and high school diplomas (33.28%) were the most common educational levels among participants. The majority of the participants did not have any children (49.63%) or reported having one (25.32%), two (20.68%), or three (3.16%) children. The majority of participants were Eastern Orthodox Christians (89.61%). Some participants identified as Catholic (4.17%), Protestant (0.74%), or Adventist (0.53%), while others reported being Baptist (0.26%), Greek Catholic (0.11%), or adhering to other religious orientations (0.90%). The sample also included 2.43% atheists, 22.89% religious but non-practicing participants, and 20.36% religious and frequent religious practitioners.

The inclusion criteria were related to age (>18 years), the absence of a disability diagnosis (to avoid bias when exploring selective POD), and no prior experience related to organ donation (i.e., the research was addressed to participants who did not report having any family member, friend, colleague, acquaintance, or other person from their close circle [or even themselves] who had been the subject of an organ donation procedure). The choice of these criteria was made to avoid bias from personal experience (participants who have previous exposure to organ donation, either via personal experience or close relationships, may have already developed beliefs, attitudes, or emotional reactions that could influence their responses to the questions of the present study). Moreover, in cases when the research pertains to delicate subjects or interventions, such as organ donation, related emotional distress or unease might also bias the responses of participants with personal experiences in organ donation. Finally, the focus on adults was also motivated by several reasons, i.e., (1) participants' legal capacity and consent, (2) autonomy and individual agency (adults are generally presumed to have the capacity to make decisions for themselves, including decisions related to organ donation), and (3) relevance to organ donation policies, which are specifically tailored for adults.

Data were collected using a paper-and-pencil format approximately 3 months prior to the onset of the COVID-19 pandemic. The sampling method used was snowball sampling, initiated through multiple channels. Initially, university students were recruited and asked to disseminate the questionnaire within their personal and professional networks. Additionally, the study was advertised via social media platforms (e.g., Facebook groups) and promoted in offline communities, including university-based student groups, as well as groups of teachers and healthcare professionals (e.g., doctors). This approach facilitated the inclusion of a diverse sample from five medium- to large-sized cities in northeast and southeast Romania. Participation was entirely voluntary, and all participants were provided with an informed consent form that emphasized their right to withdraw at any point without consequences. During the initial briefing, participants were assured that all data collected would remain anonymous and confidential and would be used exclusively for this research. Completing the questionnaire took approximately 20 min. The study complied with the ethical principles outlined in the 2013 Declaration of Helsinki and was approved by the Ethics Committee of the university with which the authors are affiliated.

### Measures

#### Beliefs associated with organ donation

The 21-item Beliefs Associated with Organ Donation Scale (O'Carroll et al., 2011) was used, and participants rated their answers on a scale ranging from 1 (strongly disagree) to 6 (strongly agree). Example items included “There is a high chance that donated organs will be trafficked on the organ black market and bought by someone who doesn't deserve them” for the *medical mistrust* subscale (α = 0.6 (95% CI [0.57, 0.63])), “I wouldn't like the idea of having someone else's organs in my body, even if I needed a transplant” for the *Ick factor* subscale (α = 0.64 (95% CI [0.61, 0.67])), “People must be buried with all their organs” for the *bodily integrity* subscale (α = 0.68 (95% CI [0.65, 0.71])), “Organ donation allows something good to remain from the tragic event of someone's death” for the *perceived benefits* subscale (α = 0.62 (95% CI [0.59, 0.65])) and “Accepting the donation is like planning my death, which would bring me bad luck” for the *Jynx factor* subscale (α = 0.7 (95% CI [0.67, 0.73])). Two items (i.e., “Through organ donation, a part of me would continue to live after my death” from perceived benefits and “Organ donors may not be able to be resurrected because they do not have all the parts” from the Jynx factor) were removed due to very low correlation with the total score. A CFA was performed, resulting in an acceptable fitted measurement model under oblique assumption with correlated factors (χ^2^ = 460.81, *df* = 48, *p* < 0.001, CFI = 0.98, TLI = 0.98, SRMR = 0.05, RMSEA = 0.07, *p* < 0.001, 90% CI [0.06, 0.07]). Higher scores indicated more negative beliefs about organ donations: higher medical mistrust, lower perceived benefits of being an organ donor, stronger beliefs related to the need to maintain bodily integrity after one's death, higher “Jynx”—i.e., bad luck related to becoming an organ donor, and higher disgust associated with the idea of organ donation (i.e., the Ick factor).

#### Attitudes toward organ donation

Attitudes toward organ donation were measured using the perceived subjective norms and perceived moral norms associated with organ donation (Hyde and White, 2009). Example items included “The people I respect would recommend that I agree to organ donation after death.” for the *subjective norm* (α = 0.65 (95% CI [0.62, 0.67])), and “I would feel guilty if I did not agree to organ donation after death in the case of a member of my family” for the *perceived moral norms* (α = 0.56 (95% CI [0.52, 0.59])). The participants rated their answers on a scale ranging from 1 (strongly disagree) to 6 (strongly agree). A CFA was performed, resulting in an acceptable fitted measurement model under the oblique assumption with correlated factors (χ^2^ = 77.17, *df* = 8, *p* < 0.001, CFI = 0.99, TLI = 0.98, SRMR = 0.04, RMSEA = 0.07, *p* = 0.015, 90% CI [0.05, 0.08]). Higher scores on subjective and perceived moral norms were associated with a more positive attitude toward organ donation.

#### Interpersonal reactivity index

We used the Interpersonal Reactivity Index (Davis, 1983a,b) to assess participants' empathy. Seven items (e.g., “Most of the time, other people's misfortunes don't bother me much” and “I often worry about people less fortunate than myself”) were answered on a 6-point Likert scale ranging from 1 (strong disagreement) to 6 (strong agreement). The total score was computed (α = 0.61 (95% CI [0.58, 0.63])). Higher scores indicated higher empathy.

#### Instrumental harm

Four items from the Oxford Utilitarianism Scale (Kahane et al., 2018) were used to assess participants' instrumental harm tendencies (α = 0.74 (95% CI [0.72, 0.76])). The participants rated their answers on a scale ranging from 1 (strongly disagree) to 6 (strongly agree). Example items included “It is morally right to harm one innocent person if it helps several other innocent people.” A CFA was performed, resulting in an acceptable fitted measurement model under the oblique assumption with correlated factors (χ^2^ = 13.81, *df* = 5, *p* = 0.017, CFI = 1, TLI = 0.99, SRMR = 0.02, RMSEA = 0.03, *p* = 0.947, 90% CI [0.01, 0.05]).

#### Intentions for posthumous organ donation—POD

We further used three single-item indicators, following the *beliefs associated with the organ donation scale* (O'Carroll et al., 2011), to assess the participants' POD in different scenarios. More specifically, we asked participants about their donation intentions in three different cases: (1) “Suppose you were put in the position of deciding on organ donation after your death. Would you agree to this intervention?” for *general* [i.e., unspecified recipient] POD; (2) “Suppose you were put in the position of deciding on organ donation after your death. Would you agree to your organs being received by a child with disabilities?” for *POD to a child with a disability*, and (3) “Suppose you were put in the position of deciding on organ donation after your death. Would you agree to your organs being received by an adult with a disability?” for *POD to an adult with disabilities*. The participants rated their answers on a scale ranging from 1 (strongly disagree) to 6 (strongly agree). Using our data, Cronbach's alpha for the total score was α = 0.92 (95% CI [0.91, 0.92]). Higher scores indicated higher agreement with organ donation in those specific scenarios.

The demographic scale included age, gender, religion, relationship status, engagement in religious practices (How often did you attend religious services or events in the last year? Never/Sometimes/Often), and the number of children.

## Results

### Overview of statistical analysis

We used R (Version 4.3.2; R Core Team, 2023) and the R packages *foreign* (Version 0.8.86; R Core Team, 2022), *kableExtra* (Version 1.4.0; Zhu, 2021), *papaya* (Version 0.1.2; Aust and Barth, 2022), *psych* (Version 2.4.1; William, 2023), and *tinylabels* (Version 0.2.4; Barth, 2022) for all our analyses. The initial assumptions assessment was conducted through descriptive univariate analysis, and data screening for outliers was performed to verify univariate normality. The Mardia indicator (Mardia, 1970) was computed to assess multivariate normality. Internal consistency was assessed using Cronbach's α indicator (Cronbach, 1951), and a confirmatory factor analysis based on diagonally weighted least squares (DiStefano and Morgan, 2014) was used to test the instruments' factorial validity and dimensional structure.

A moderated SEM model was assessed using robust SEM techniques, and parameters were estimated. For *interaction terms*, we defined the upstream latent variables “Beliefs about organ donation” and “Attitude toward organ donation” by setting up and assessing the measurement model. The predicted values for the latent variables were then extracted and added to the dataset. Then, we centered the predicted scores for the latent variables “Beliefs about organ donation” and “Theory of planned behavior factors” by their grand mean and the observed scores for the moderators “empathic concern” and “instrumental harm.” Products between each centered upstream variable and centered moderator variable were computed, resulting in the interaction terms. The effect of seven categorical variables was controlled for, including them as covariates in the model (i.e., education, marital status, number of children, religion, religious practitioner, gender, and age). Finally, four alternative, non-hierarchical SEM models were assessed. The first *model* included only main effects and covariates; the second *model* added only the interaction terms of empathic concern; the *third model* added only the interaction terms of utilitarianism; and the *fourth model* was the full moderated model. The models' comparison was performed using the LRT test to compare nested models (Satorra, 2000).

### Preliminary analysis

An initial descriptive analysis was conducted to assess the univariate normality assumptions for the scalar variables. Our data suggested that the univariate normality assumption was not met for any of the continuous variables in the model (see Table 1). The assumption of multivariate normality based on the Mardia coefficient (Mardia, 1970) was not met. Our results suggested Mahalanobis distances from the centroid coordinates ranging from 0.94 to 9.45, along with a statistically significant positive multivariate skewness (Mardia = 7.12, Skewness = 2.240.39, *p* < 0.001) and multivariate leptokurtic distribution (Mardia = 148.96, Skewness = 40.61, *p* < 0.001).

**Table 1:** Preliminary descriptive analysis and normality assumption assessment.

**Variables**	**Mean**	**SD**	**Min**	**Max**	**Skew (SE)**	**Kurt (SE)**	**Shapiro-Wilk (*p*)**
Medical mistrust	17.60	4.73	5	30	0.01 (0.06)	−0.32 (0.11)	0.99 (< 0.001)
The Ick factor	7.05	3.58	3	18	0.66 (0.06)	−0.41 (0.11)	0.91 (< 0.001)
Bodily integrity	4.69	2.48	2	12	0.81 (0.06)	−0.01 (0.11)	0.90 (< 0.001)
Perceived benefit	14.90	2.82	3	21	−0.97 (0.06)	0.69 (0.11)	0.90 (< 0.001)
The Jynx factor	4.06	2.43	2	18	1.16 (0.06)	0.94 (0.11)	0.82 (< 0.001)
Subjective norms	12.12	3.03	3	19	−0.17 (0.06)	−0.14 (0.11)	0.98 (< 0.001)
Perceived moral norms	11.60	2.33	3	18	−0.28 (0.06)	0.46 (0.11)	0.98 (< 0.001)
Empathy	24.15	3.83	10	38	0.20 (0.06)	1.13 (0.11)	0.98 (< 0.001)
Instrumental harm	10.56	4.45	4	24	0.35 (0.06)	−0.40 (0.11)	0.96 (< 0.001)

#### Correlation analysis

The majority of Spearman's ρ correlations were statistically significant (see Table 2), with values between −0.59 and 0.65, and the correlation matrix was positively defined. *Medical mistrust* was positively associated with all the dimensions of beliefs about organ donation (all *p*-s < 0.05), subjective norms (ρ = −0.178, *p* < 0.001), perceived moral norms (ρ = −0.121, *p* < 0.001), empathy (ρ = 0.059, *p* = 0.01), instrumental harm (ρ = 0.054, *p* = 0.02), and POD intentions (ρ = −0.293, *p* < 0.001). *The Ick factor* was positively associated with bodily integrity (ρ = 0.653, *p* < 0.001) and the Jynx factor (ρ = 0.649, *p* < 0.001), and negatively associated with perceived benefits (ρ = −0.484, *p* < 0.001), subjective norms (ρ = −0.381, *p* < 0.001), perceived moral norms (ρ = −0.3, *p* < 0.001) and POD intentions (ρ = −0.59, *p* < 0.001). *Bodily integrity* was positively associated with the Jynx factor (ρ = 0.616, *p* < 0.001), instrumental harm (ρ = 0.046, *p* = 0.047), and negatively associated with perceived benefits (ρ = −0.509, *p* < 0.001), subjective norms (ρ = −0.389, *p* < 0.001), perceived moral norms (ρ = −0.331, *p* < 0.001), and POD (ρ = −0.59, *p* < 0.001). *Perceived benefits* were positively associated with subjective norms (ρ = 0.388, *p* < 0.001), perceived moral norms (ρ = 0.387, *p* < 0.001), empathy (ρ = 0.054, *p* = *0.019*), instrumental harm (ρ = 0.048, *p* = *0.037*), and POD (ρ = 0.46, *p* < 0.001), and negatively associated with the Jynx factor (ρ = −0.481, *p* < 0.001). *The Jynx factor* was positively associated with empathy (ρ = 0.085, *p* < 0.001), instrumental harm (ρ = 0.057, *p* = 0.013), and negatively associated with subjective norms (ρ = −0.332, *p* < 0.001), perceived moral norms (ρ = −0.245, *p* < 0.001), and POD (ρ = −0.53, *p* < 0.001). *Subjective norms* were positively associated with perceived moral norms (ρ = 0.362, *p* < 0.001), empathy (ρ = 0.05, *p* = *0.03*), instrumental harm (ρ = 0.161, *p* < 0.001), and POD intentions (ρ = 0.45, *p* < 0.001). *Perceived moral norms* were positively associated with instrumental harm (ρ = 0.095, *p* < 0.001) and POD intentions (ρ = 0.37, *p* < 0.001). *Empathy* was positively associated with instrumental harm (ρ = 0.134, *p* < 0.001), and *instrumental harm* was positively associated with POD intentions (ρ = 0.06, *p* = 0.009).

**Table 2:** Spearman correlation matrix (Cronbach's alpha on the main diagonal).

**Variable**	**1**	**2**	**3**	**4**	**5**	**6**	**7**	**8**	**9**	**10**
(1) Medical mistrust	0.600									
(2) The Ick factor	0.369^***^	0.640								
(3) Bodily integrity	0.315^***^	0.653^***^	0.680							
(4) Perceived benefit	−0.183^***^	−0.484^***^	−0.509^***^	0.620						
(5) The Jynx factor	0.308^***^	0.649^***^	0.616^***^	−0.481^***^	0.700					
(6) Subjective norms	−0.178^***^	−0.381^***^	−0.389^***^	0.388^***^	−0.332^***^	0.560				
(7) Perceived moral norms	−0.121^***^	−0.300^***^	−0.331^***^	0.387^***^	−0.245^***^	0.362^***^	0.560			
(8) Empathic concern	0.059^*^	0.036	0.040	0.054^*^	0.085^***^	0.050^*^	0.028	0.610		
(9) Instrumental harm	0.054^*^	0.022	0.046^*^	0.048^*^	0.057^*^	0.161^***^	0.095^***^	0.134^***^	0.730	
(10) Donation intentions	−0.293^***^	−0.590^***^	−0.590^***^	0.467^***^	−0.534^***^	0.455^***^	0.373^***^	−0.007	0.060^**^	0.920
Means	17.603	7.047	4.691	14.902	4.057	12.119	11.599	24.152	15.695	14.335
Standard deviations	4.728	3.585	2.481	2.817	2.432	3.034	2.325	3.829	4.902	3.877

^*^p ≤ 0.050; ^**^p ≤ 0.010; ^***^p ≤ 0.001.

### Model analysis

Since the assumption of multivariate normality was not fulfilled, all SEM models were estimated using the DWLS method (DiStefano and Morgan, 2014). Only the main effects of the upstream variables, moderators, and covariates were included in the first model, and convergence was achieved after 224 iterations, estimating 66 parameters based on 1,813 cases. An over-identified model resulted in acceptable fit indices (χ^2^ = 366.183, *df* = 70, *p* < 0.001, CFI = 0.974, SRMR = 0.041, RMSEA = 0.048, *p* = 1, 90% CI [0.044, 0.053]; see Table 3).

**Table 3:** Global fit indicators and fit indexes.

**Iterations**	**Parameters**	**χ^2^ (*p*)**	***df***	**CFI**	**SRMR**	**RMSEA (*p*)**	**95% CI**
**I. Main effects with covariates**							
224	66	366.183 (< 0.001)	70	0.974	0.041	0.048 (0.705)	0.044 – 0.053
**II. Main effects moderated by empathic concern**							
267	87	393.073 (< 0.001)	94	0.973	0.039	0.045 (0.963)	0.041 – 0.050
**III. Main effects moderated by utilitarianism**							
216	87	500.42 (< 0.001)	84	0.964	0.045	0.052 (0.190)	0.048 – 0.057
**IV. Main effects moderated by utilitarianism**							
244	76	470.357	77	0.966	0.046	0.053 (0.130)	0.049 – 0.058

#### General POD

General POD (i.e., no other specifiers). Our initial results suggested a significant main association between general POD and beliefs about organ donation (*B* = −0.32, *z* = −6.02, *p* < 0.001, β = −0.43), as well as between the TPB factors and general POD (*B* = 0.27, *z* = 4.37, *p* < 0.001, β = 0.39). Higher scores on general POD were associated with lower scores on beliefs about organ donation and higher scores on the TPB factors. Among the moderator variables, only instrumental harm was significantly associated with general POD (*B* = −0.01, *z* = −2.39, *p* = 0.017, β = −0.08): higher scores on general POD were associated with lower instrumental harm. No significant association was observed between general POD and empathic concern (*B* = 0.02, *z* = 2.12, *p* = 0.034, β = 0.06).

#### Donation intentions for a child with disabilities

Furthermore, higher donation intentions to a child with disabilities were significantly associated with lower scores on beliefs about organ donation (*B* = −0.30, *z* = −6.24, *p* < 0.001, β = −0.43) and higher scores on the TPB factors (*B* = 0.20, *z* = 3.65, *p* < 0.001, β = 0.30). In contrast, as a moderator variable, empathic concern was not significantly associated with the dependent variable (*B* = 0.01, *z* = 0.85, *p* = 0.398, β = 0.03). However, instrumental harm was negatively associated with POD to a child with disabilities (*B* = −0.03, *z* = −3.09, *p* = 0.002, β = = −0.09).

#### Donation intentions toward an adult with disabilities

Next, participants' scores on beliefs about organ donation (*B* = −0.31, *z* = −6.47, *p* < 0.001, β = −0.43) and the TPB factors (*B* = 0.20, *z* = 3.69, *p* < 0.001, β = 0.30) were also significantly associated with POD intentions for an adult with disabilities: higher scores on POD intentions for an adult with disabilities were associated with lower scores on beliefs about organ donation and higher scores on the TPB factors. empathic concern was not significantly associated with the dependent variable (*B* = 0.00, *z* = 0.36, *p* = 0.718, β = 0.01), but instrumental harm was negatively associated (*B* = −0.03, *z* = −3.60, *p* < 0.001, β = −0.11).

#### Controlled variables

**Figure 2:**
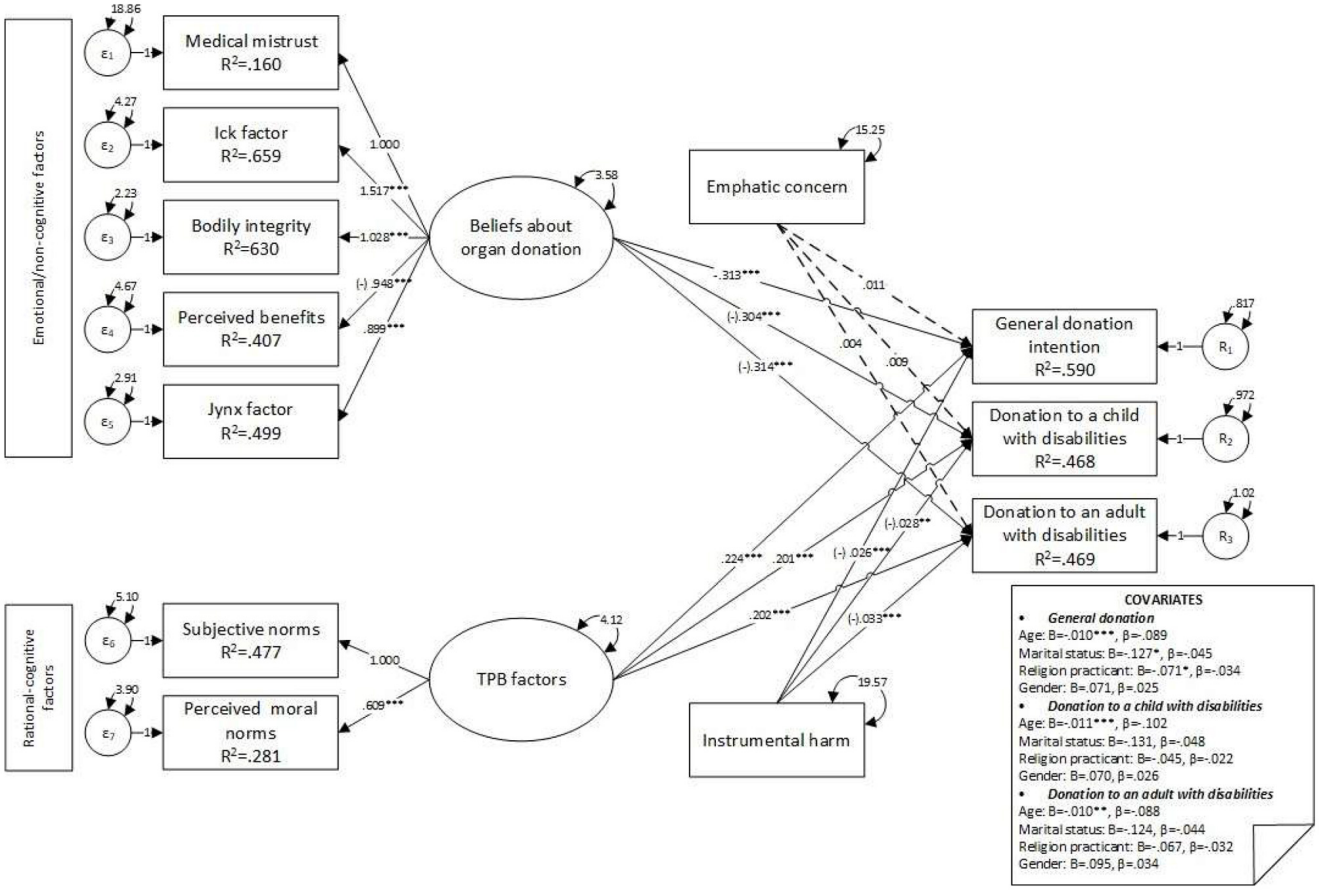
Model 1—main effects of upstream and moderator variables on donation intention factors. *p ≤ 0.050; **p ≤ 0.010; ***p ≤ 0.001.

Four variables were controlled for all three dependent variables: age, marital status, religious practices, and gender, and the results indicated statistically significant effects. Age was negatively associated with general POD (*B* = −0.01, *z* = −2.39, *p* = 0.017, β = −0.08), POD to a child with disabilities” (*B* = −0.01, *z* = −2.93, *p* = 0.003, β = −0.10), and POD to an adult with disabilities (*B* = −0.01, *z* = −2.62, *p* = 0.009, β = −0.09): as age increased, scores on all three dependent variables decreased. Marital status (*B* = −0.13, *z* = −1.34, *p* = 0.179, β = −0.04) and religious practices (*B* = −0.10, *z* = −1.88, *p* = 0.06, β = −0.05) were significantly associated only with general POD intentions. No significant associations were observed regarding participants' gender (see Table 4 and Figure 2).

**Table 4:** Model 1—primary effects of upstream and moderator variables on POD.

**Outcomes**	**Predictors**	**Estimator**	**SE**	***z***	***p***	**Beta**
General donation < -	Beliefs	−0.32	0.05	−6.02	< 0.001	−0.43
	TPB factors	0.27	0.06	4.37	< 0.001	0.39
	Empathic concern	0.02	0.01	2.12	=0.034	0.06
	Instrumental harm	−0.02	0.01	−1.88	=0.060	−0.05
Donation to a child < -	Beliefs	−0.30	0.05	−6.24	< 0.001	−0.43
	TPB factors	0.20	0.06	3.65	< 0.001	0.30
	Empathic concern	0.01	0.01	0.85	=0.398	0.03
	Instrumental harm	−0.03	0.01	−3.09	=0.002	−0.09
Donation to an adult < -	Beliefs	−0.31	0.05	−6.47	< 0.001	−0.43
	TPB factors	0.20	0.05	3.69	< 0.001	0.30
	Empathic concern	0.00	0.01	0.36	=0.718	0.01
	Instrumental harm	−0.03	0.01	−3.60	< 0.001	−0.11

**Figure 3:**
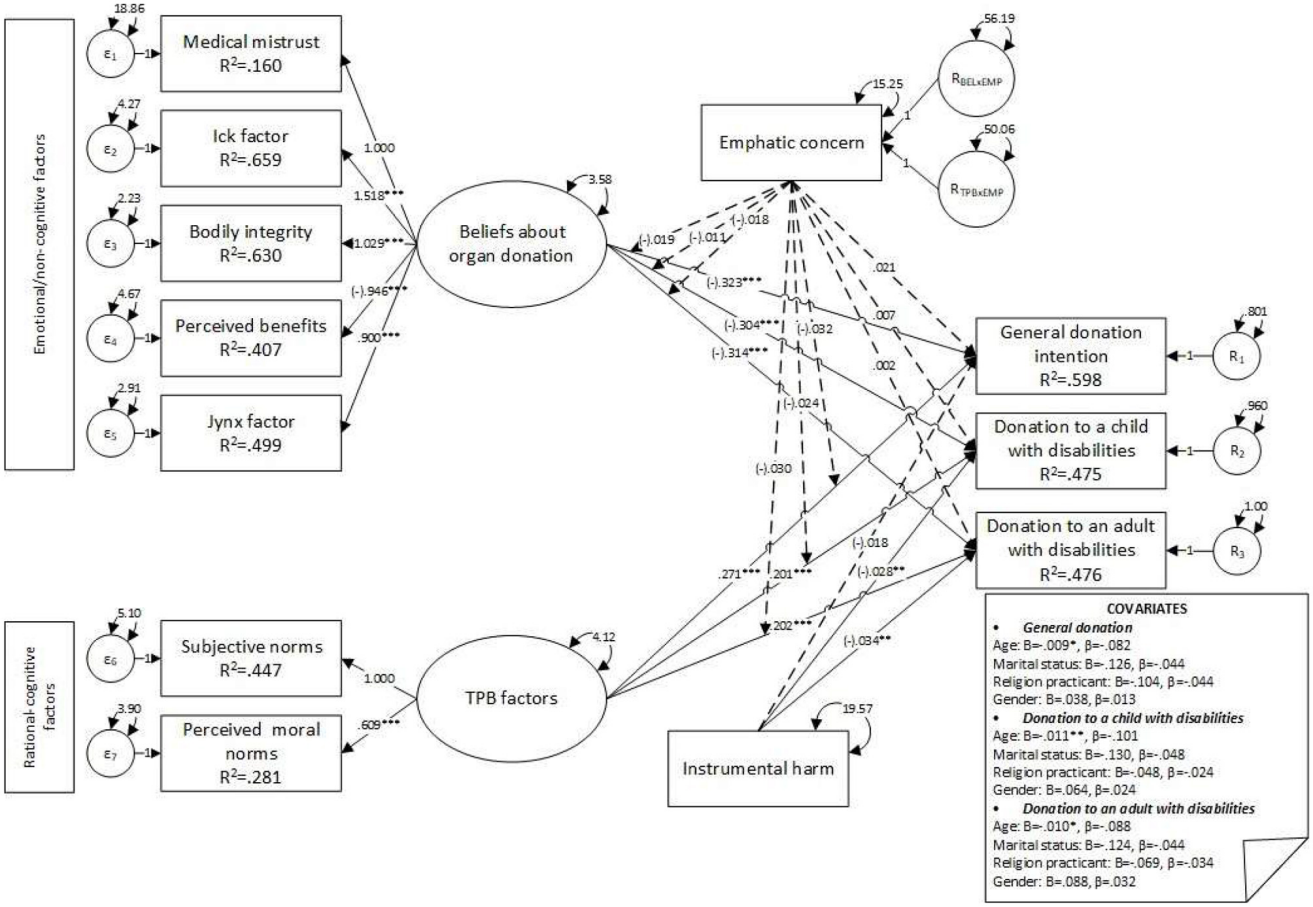
Model 2—main effects on factors influencing donation intentions, moderated by empathic concern. *p ≤ 0.050; **p ≤ 0.010; ***p ≤ 0.001.

Interaction terms for empathic concern were added to the first model, and the second model was specified (see Figure 3). Convergence was achieved after 267 iterations, estimating 87 parameters based on 1,813 cases. An over-identified model resulted in acceptable fit indices (χ^2^ = 393.073, *df* = 84, *p* < 0.001, CFI = 0.973, SRMR = 0.039, RMSEA = 0.045, *p* = 0.963, 90% CI [0.041, 0.05]). Significant differences were observed in the fit between the first and the second model ($\chi_{\text{diff}}^{2}$ = 26.89, *df*_diff_ = 14, *p* = 0.02, RMSEA = 0.02), and the first model was better sustained by data than the second. No moderation effects of empathic concern were observed, and the main association between empathic concern and POD factors was not significant (see Table 5). Moreover, the negative main association between instrumental harm and general POD decreased, and statistical significance was lost (*B* = −0.02, *z* = −1.83, *p* = 0.068, β = −0.06). A negative covariance was observed between age and all three dependent variables (see Figure 3); no other significant covariances were observed.

**Table 5:** Model 2—main effects on factors influencing donation intentions moderated by empathic concern.

**Outcomes**	**Predictors**	**Estimator**	**SE**	***z***	***p***	**Beta**
General donation < -	Beliefs	−0.32	0.05	−6.02	< 0.001	−0.43
	TPB factors	0.27	0.06	4.37	< 0.001	0.39
	Empathic concern	0.02	0.01	1.72	=0.086	0.06
	Instrumental harm	−0.02	0.01	−1.83	=0.068	−0.06
	Beliefs × Empathic concern	−0.02	0.04	−0.48	=0.630	−0.10
	Planned behavior × Empathic concern	−0.03	0.04	−0.76	=0.447	−0.16
Donation to a child < -	Beliefs	−0.30	0.05	−6.24	< 0.001	−0.43
	TPB factors	0.20	0.06	3.65	< 0.001	0.30
	Empathic concern	0.01	0.01	0.57	=0.571	0.02
	Instrumental harm	−0.03	0.01	−2.99	=0.003	−0.09
	Beliefs × Empathic concern	−0.01	0.04	−0.29	=0.769	−0.06
	Planned behavior × Empathic concern	−0.02	0.04	−0.64	=0.521	−0.13
Donation to an adult < -	Beliefs	−0.31	0.05	−6.47	< 0.001	−0.43
	TPB factors	0.20	0.05	3.69	< 0.001	0.30
	Empathic concern	0.00	0.01	0.21	=0.837	0.01
	Instrumental harm	−0.03	0.01	−3.48	< 0.001	−0.11
	Beliefs × Empathic concern	−0.02	0.04	−0.47	=0.639	−0.10
	Planned behavior × Empathic concern	−0.03	0.04	−0.76	=0.449	−0.15

**Figure 4:**
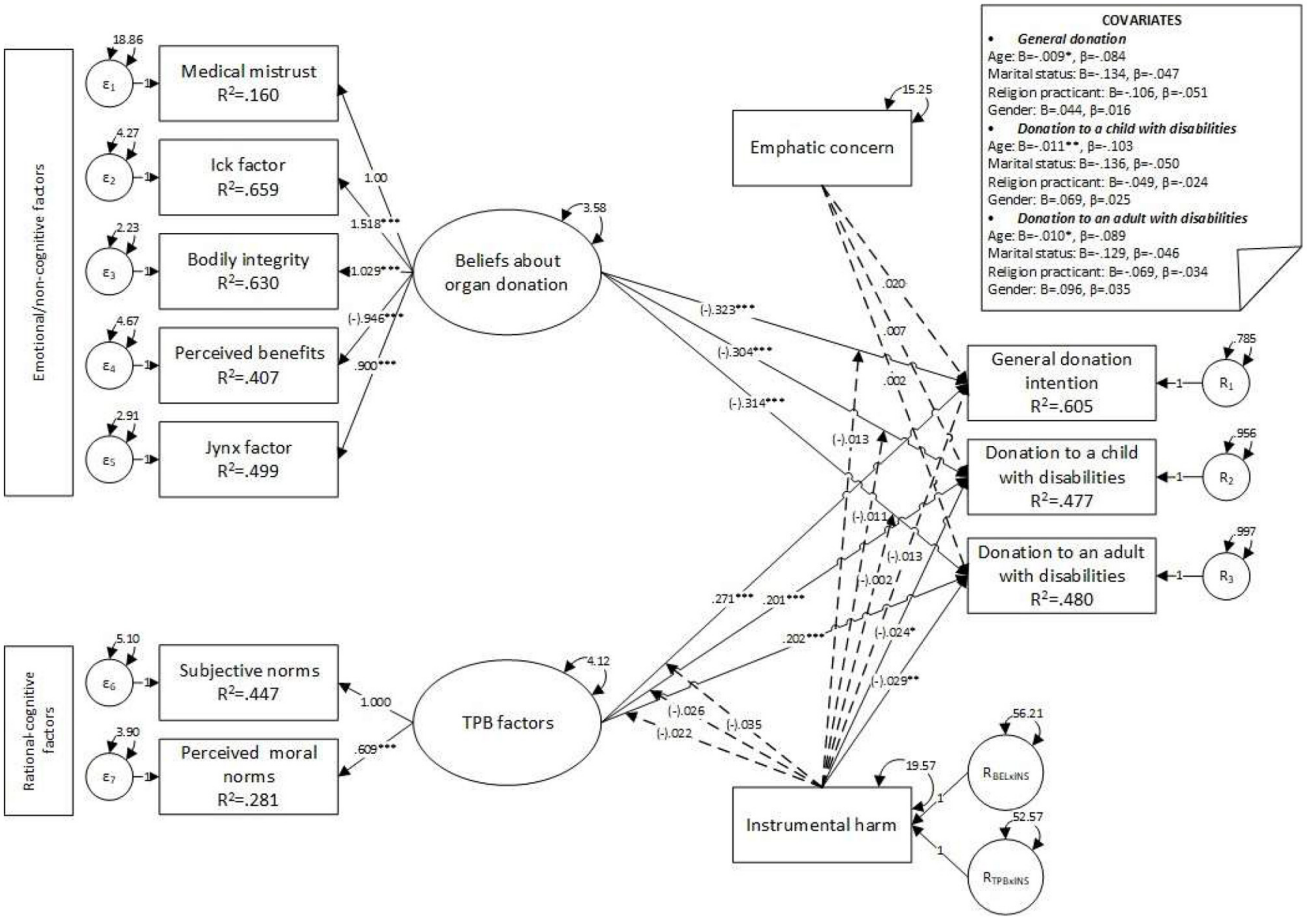
Model 3—main effects on donation intention factors moderated by instrumental harm. *p ≤ 0.050; **p ≤ 0.010; ***p ≤ 0.001.

Interaction terms for empathic concern were removed, and interaction terms for instrumental harm were added to the first model (see Figure 4). Convergence was achieved after 216 iterations, estimating 87 parameters based on 1,813 cases. An over-identified model resulted, with acceptable fit indices (χ^2^ = 500.42, *df* = 84, *p* < 0.001, CFI = 0.964, SRMR = 0.045, RMSEA = 0.052, *p* = 0.19, 90% CI [0.048, 0.057]), and the model showed a statistically significant poorer fit than the first model ($\chi_{\text{diff}}^{2}$ = 134.24, *df*_diff_ = 14, *p* < 0.001, RMSEA = 0.07); no moderation effect of instrumental harm was observed. Instrumental harm was negatively associated with POD to a child with disabilities (*B* = −0.02, *z* = −2.59, *p* = 0.01, β = −0.08) and POD to an adult with a disability (*B* = −0.03, *z* = −2.98, *p* = 0.003, β = −0.09). Moreover, no association with general POD (*B* = −0.01, *z* = −1.28, *p* = 0.201, β = −0.04) was observed (see Table 6). Furthermore, results indicated negative significant covariances between age and general POD (*B* = −0.01, *z* = −2.40, *p* = 0.016, β = −0.08), POD to a child with disabilities (*B* = −0.01, *z* = −2.94, *p* = 0.003, β = −0.10), and POD to an adult with disabilities (*B* = −0.01, *z* = −2.64, *p* = 0.008, β = −0.09).

**Table 6:** Model 3—main effects on donation intentions' factors moderated by instrumental harm.

**Outcomes**	**Predictors**	**Estimator**	**SE**	***z***	***p***	**Beta**
General donation < -	Beliefs	−0.32	0.05	−6.02	< 0.001	−0.43
	TPB factors	0.27	0.06	4.37	< 0.001	0.39
	Empathic concern	0.02	0.01	1.82	=0.069	0.05
	Instrumental harm	−0.01	0.01	−1.28	=0.201	−0.04
	Beliefs × Instrumental harm	−0.01	0.03	−0.52	=0.605	−0.07
	Planned behavior × Instrumental harm	−0.03	0.03	−1.30	=0.194	−0.18
Donation to a child < -	Beliefs	−0.30	0.05	−6.24	< 0.001	−0.43
	TPB factors	0.20	0.06	3.65	< 0.001	0.30
	Empathic concern	0.01	0.01	0.66	=0.510	0.02
	Instrumental harm	−0.02	0.01	−2.59	=0.010	−0.08
	Beliefs × Instrumental harm	−0.01	0.02	−0.42	=0.672	−0.06
	Planned behavior × Instrumental harm	−0.03	0.03	−1.00	=0.315	−0.14
Donation to an adult < -	Beliefs	−0.31	0.05	−6.47	< 0.001	−0.43
	TPB factors	0.20	0.05	3.69	< 0.001	0.30
	Empathic concern	0.00	0.01	0.14	=0.885	0.00
	Instrumental harm	−0.03	0.01	−2.98	=0.003	−0.09
	Beliefs × Instrumental harm	0.00	0.02	−0.08	=0.935	−0.01
	Planned behavior × Instrumental harm	−0.02	0.03	−0.87	=0.384	−0.12

**Figure 5:**
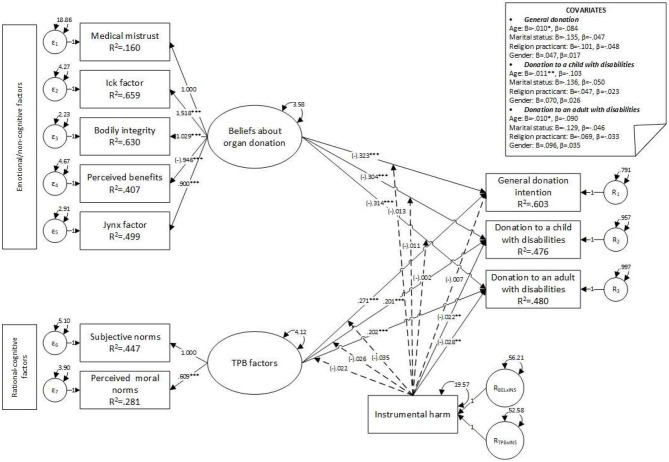
Model 4—main effects on factors influencing donation intentions moderated by instrumental harm. Unstandardized coefficients. *p ≤ 0.050; **p ≤ 0.010; ***p ≤ 0.001.

**Figure 6:**
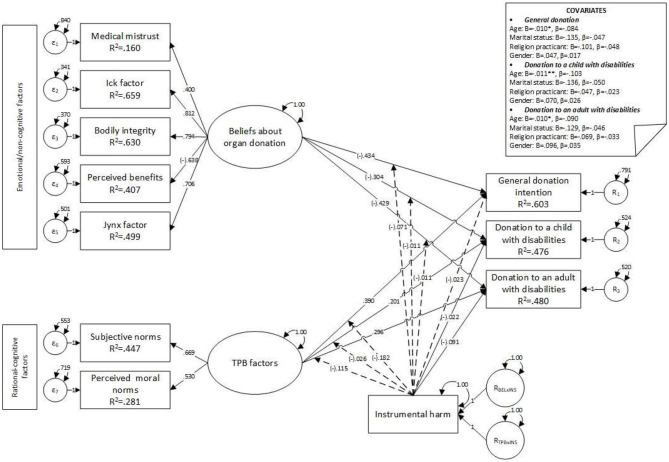
Model 4—main effects on donation intentions, with factors moderated by instrumental harm. Standardized coefficients. *p ≤ 0.050; **p ≤ 0.010; ***p ≤ 0.001.

The model with interaction terms for empathic concern and instrumental harm yielded an unstable solution for all three dependent variables, as the iteration criterion was exceeded (over 10,000 iterations). The model was respecified by removing the empathic concern (see Figures 5, 6), and the final parameters were estimated. Convergence was achieved after 244 iterations, estimating 76 parameters based on 1,813 cases, and an over-identified model resulted, with acceptable fit indices (χ^2^ = 470.357, *df* = 77, *p* < 0.001, CFI = 0.966, SRMR = 0.046, RMSEA = 0.053, *p* = 0.13, 90% CI [0.049, 0.058]). No statistically significant covariates were observed, and the main associations were preserved, including the negative association between instrumental harm and POD. No interaction effects were observed (see Table 7 and Figures 5, 6).

**Table 7:** Model 4—main effects on factors influencing donation intentions moderated by instrumental harm.

**Outcomes**	**Predictors**	**Estimator**	**SE**	***z***	***p***	**Beta**
General donation < -	Beliefs	−0.32	0.05	−6.02	< 0.001	−0.43
	TPB factors	0.27	0.06	4.37	< 0.001	0.39
	Instrumental harm	−0.01	0.01	−0.85	=0.395	−0.02
	Beliefs × Instrumental harm	−0.01	0.03	−0.52	=0.606	−0.07
	Planned behavior × Instrumental harm	−0.04	0.03	−1.32	=0.186	−0.18
Donation to a child < -	Beliefs	−0.30	0.05	−6.24	< 0.001	−0.43
	TPB factors	0.20	0.06	3.65	< 0.001	0.30
	Instrumental harm	−0.02	0.01	−2.75	=0.006	−0.07
	Beliefs × Instrumental harm	−0.01	0.02	−0.42	=0.672	−0.06
	Planned behavior × Instrumental harm	−0.03	0.03	−1.01	=0.310	−0.14
Donation to an adult < -	Beliefs	−0.31	0.05	−6.47	< 0.001	−0.43
	TPB factors	0.20	0.05	3.69	< 0.001	0.30
	Instrumental harm	−0.03	0.01	−3.38	< 0.001	−0.09
	Beliefs × Instrumental harm	0.00	0.02	−0.08	=0.935	−0.01
	Planned behavior × Instrumental harm	−0.02	0.03	−0.87	=0.382	−0.12

Finally, means were computed, and multiple comparisons were conducted using an ordinal regression model to assess whether there was a significant difference between different donation intentions. General POD (*m* = 4.73) was significantly lower than POD to a child with disabilities [*m* = 4.86, $\chi_{\left(5\right)}^{2}$ = 96.86, *p* < 0.001] and POD to an adult with disabilities [*m* = 4.74, $\chi_{\left(5\right)}^{2}$ = 233.02, *p* < 0.001]. Moreover, POD to a child with disabilities (*m* = 4.86) was significantly higher than POD to an adult with disabilities [*m* = 4.74, $\chi_{\left(5\right)}^{2}$ = 1,231.86, *p* < 0.001].

## Discussions

The present study examined some of the factors related to adults' intentions to donate organs after their death. We investigated the roles of the beliefs associated with organ donation, perceived subjective and moral norms, empathic concern, and instrumental harm concerning general POD intentions and two specific scenarios involving donation to a child or an adult with disabilities (i.e., selective organ donation).

We hypothesized that beliefs about organ donation would be significantly associated with POD intentions. Results indicated that perceived benefits were positively associated with POD intentions, while bodily integrity, the Ick and Jynx factors, and medical mistrust were negatively associated with POD intentions. These results are consistent with previous findings (Williamson et al., 2019; Pfaller et al., 2018; Bramstedt, 2022; Clark et al., 2023; Alsalem et al., 2020), suggesting that individuals who do not trust the medical system and perceive it as incompetent and unable to help those in need might be less likely to sign up as organ donors (Williamson et al., 2019). Similarly, individuals who are concerned about preserving the dignity of deceased individuals might oppose organ donation to preserve the bodies of the deceased (Pfaller et al., 2018). Furthermore, individuals might oppose organ donation due to the intense disgust associated with the process of transplanting organs (Bramstedt, 2022) or due to various superstitions and spiritual beliefs associated with adverse events that might be associated with organ recitation (Clark et al., 2023). However, when individuals perceive that those in need of medical assistance might benefit from receiving donated organs, they may be more willing to sign up as potential donors (Alsalem et al., 2020).

Next, we assumed that the TPB factors—i.e., subjective and perceived moral norms—would be significantly associated with POD intentions. Results indicated that subjective and perceived moral norms were positively associated with POD intentions. These results converge with previous findings, which suggested the relevance of the TPB factors in explaining POD intentions (Manstead, 2000). In line with the TPB framework, our findings suggest that, in their decision to donate their organs posthumously, individuals might be strongly influenced by the attitudes toward organ donation adopted by their families, friends, peers, and other important persons in their social circles (i.e., subjective norms) and by the perceived righteousness of organ donation (i.e., perceived moral norms; Hyde and White, 2009). Moreover, subjective norms and perceived moral norms also predicted POD intentions for patients with disabilities. These results suggest that, in general, individuals who perceive organ donation as a moral act that is also endorsed by other individuals important to them might be willing to posthumously donate their organs, regardless of the specifics of the patients in need. These findings are relevant since they address the gap in research on the role of TPB in explaining POD intentions.

Furthermore, we assumed that empathic concern and instrumental harm would be significantly associated with POD intentions and would moderate the link between beliefs about organ donation, TPB factors, and POD intentions. Results indicated that empathic concern did not significantly influence POD intentions in any investigated scenarios. These results appear to diverge from previous findings in the available literature, which have previously highlighted the predictive role of empathy regarding POD intentions (Wilczek-Rużyczka et al., 2014). However, previous research also suggested that empathy might be a more relevant predictor of the beliefs related to the advantages and costs of organ donation without necessarily having a significant effect on the attitudes related to the morality or social responsibility of organ donation (Rodrigue et al., 2004). The results of the present study might also suggest that empathy does not necessarily play a significant role in predicting POD intentions. While some individuals might empathize with those in need of donated organs, others might also empathize with the potential donors, being concerned about their integrity, even after their death. Nevertheless, further research is necessary to validate these claims.

Instrumental harm was negatively associated with general POD intentions, POD to a child, and POD to an adult with disabilities. These findings suggest that individuals who exhibit high levels of instrumental harm might be less willing to donate their organs posthumously. These results appear counterintuitive, considering the previous studies that suggested that individuals who follow utilitarian principles might be more supportive of organ donation (Shin et al., 2022). However, these results might also be explained by the complexities of the organ donation process. For example, some previous studies suggested that in some cases of organ donation (e.g., patients with acute liver failure in need of donors), individuals might be less willing to donate due to low perceived benefits (i.e., in these instances, the success chance of the operation is relatively low, and therefore individuals prefer to refuse to donate; Volk et al., 2008). Moreover, when considering the scenarios involving patients with disabilities, participants with higher instrumental harm might consider that other patients should have priority in receiving the organs if needed. However, further research is needed to better understand the role that instrumental harm (and utilitarian tendencies, in general) might play in influencing POD intentions.

Finally, we examined potential differences regarding POD intentions when discussing selective organ donation. We assumed that POD intentions would significantly differ depending on the recipient's general POD—i.e., no specifiers, POD to a child with disabilities, or POD to an adult with disabilities—with the lowest rate being reported when the recipient is an adult with a disability. The results did not confirm this assumption: the findings suggested that participants' POD intentions was the highest when discussing donation to a child with disabilities and the lowest when expressing general POD intentions. These results suggest that our participants might have prioritized donating to children with disabilities due to a higher perceived vulnerability. The current research literature suggests that individuals might prefer prioritizing organ donation to those seen as more vulnerable, favoring children over adults (Murphy and Veatch, 2006). General POD intentions may not have provided sufficient information about the specific needs of potential patients. Moreover, individuals might be reluctant to donate organs when they are concerned about the potential risks that patients they deem as undeserving might receive the organs (Morgan et al., 2008a). However, further research might help us better understand the importance of knowing some details about the recipient and other complex mechanisms that may shape selective organ donation, particularly in the case of recipients with disabilities. Moreover, an interesting future research direction might be related to how individuals without disabilities feel about organ donation to/or receiving from individuals with disabilities through the lens of out-group bias theories (Babik and Gardner, 2021).

### Implications

The results of the current study hold various practical implications. First, to encourage individuals to be more open to posthumous organ donation, efforts should be made to increase their trust in the medical system and to acknowledge the importance of organ donation in saving human lives. We are already aware that trust in medicine and the medical staff plays a crucial role in the organ donation process (Almassi, 2014), and the current results sustain these previous suggestions. Therefore, providing the public with information and guidance about organ donation might encourage individuals to sign up as posthumous donors. This factor may be particularly relevant for Romanians, as their reluctance to donate organs appears to be strongly influenced by a lack of trust in the medical system (Grigoras et al., 2010).

Moreover, spiritual beliefs related to the deceased (i.e., bodily integrity and the Jynx factor) might represent a significant barrier to posthumous organ donation. Furthermore, considering the cultural context, it is worth noting that most of our participants identified as religious individuals who engaged in frequent religious practices. Thus, some of them might be particularly concerned about the potential negative religious implications of organ donation (Bapat and Kedlaya, 2010). Therefore, presenting the process of organ transplantation as beneficial and dignifying for the donors might positively influence POD intentions. Nevertheless, these directions need to be explored in further studies that would benefit from considering the role of religious beliefs in this regard, religious practices, death rituals, and so on, especially in Romania—one of the most religious countries in Europe (Evans and Baronavski, 2018), and the Romanian Church is one of the most trusted institutions (with 70% confidence; Maftei et al., 2023). The Romanian Orthodox Church does not oppose organ donation. However, it promotes it only under specific ethical conditions, such as informed consent, the absence of financial gain, and respect for the body as a sacred creation (Romanian Patriarchate, 2009): “The Church does not prohibit anything from anyone but only invites discernment and placing any gesture or action in the context of moral conscience, ultimately concerned with the life and dignity of the human person. The removal/donation of a living organ from a person who is brain dead (and therefore incomplete) in order to save the life of another person can be such a gesture” (Bnescu, 2023).

Second, subjective norms and perceived moral norms appear to play an important role in predicting participants' POD intentions: individuals might be more willing to donate their organs when people in their social circles also approve of organ transplantation. Moreover, POD intentions appear to be strongly influenced by convictions related to the moral duty to donate. These findings suggest the relevance of promoting collective responsibility in the organ donation process. Thus, campaigns promoting posthumous organ donation as a collective effort to improve society might increase the overall willingness to donate organs (Hansen et al., 2018).

Finally, the current results suggest that the effects of empathy and instrumental harm on POD intentions may not be as direct as previously suggested. Therefore, various factors that might influence the effect of these variables should be further investigated. Utilitarian individuals might be driven not only by pursuing options that benefit most individuals but also by evaluating the efficiency of sacrificial decisions in the context of organ donation. Thus, while utilitarian decisions are often encountered in medical contexts (Georgiadou et al., 2015), other factors must also be considered when examining POD intentions in the general population (e.g., perceived chances of the intervention's success). Moreover, empathy may be more relevant in determining whether individuals identify more benefits in organ donation rather than perceiving any moral responsibility toa donate (Rodrigue et al., 2004).

### Limitations and future research directions

Several limitations of the current study need to be addressed. First, we used a convenience sample, which limits the generalizability of the present findings. To address this limitation, future studies might use more representative samples that better reflect the characteristics of the investigated population. Second, we operationalized our variables by using self-reported questionnaires. This decision increased the risk of desirability bias (i.e., participants offering socially desirable answers). Future studies should consider employing experimental designs to obtain more objective data on the investigated variables, thereby addressing this limitation. Another important limitation of the current study is the focus on a limited number of scenarios that presented very limited information (i.e., general organ donation and donating to children and adults with disabilities). Previous research highlighted the role of other factors that might influence POD intentions, such as perceived similarity to the receiving patient (Kong and Lee, 2021) and perceived chances of success for the medical procedure (Volk et al., 2008). Therefore, future studies may benefit from implementing various scenarios and considering other variables that influence POD intentions and selective organ donation.

## Conclusion

The current study aimed to investigate the role of beliefs about organ donation, subjective and moral norms, and the characteristics of potential recipients (i.e., adults and children with disabilities) in influencing organ donation while also accounting for the potential moderating role of empathic concern and instrumental harm. The results highlighted the relevance of beliefs about organ donation and the role of some of the TPB factors in influencing participants' POD intentions. While empathy did not play an important role in this regard, instrumental harm unexpectedly decreased participants' POD intentions. These findings highlight the complexity of the factors and mechanisms underlying POD intentions and selective organ donation and the need to explore societal attitudes toward organ donation further to promote a collective effort to normalize posthumous organ donation. Moreover, the current results highlight the need for further research to better understand the role of utilitarian tendencies in predicting POD intentions and selective organ donation.

## Data Availability

The raw data supporting the conclusions of this article will be made available by the authors, without undue reservation.

## References

[B1] Ajzen I. (1991). The theory of planned behavior. Organ. Behav. Hum. Decis. Process. 50, 179–211. 10.1016/0749-5978(91)90020-T

[B2] Almassi B. (2014). Trust and the duty of organ donation. Bioethics 28, 275–283. 10.1111/bioe.12096 24731143

[B3] Alsalem A. Fry M.-L. Thaichon P. (2020). To donate or to waste it: understanding posthumous organ donation attitude. Austral. Market. J. 28, 87–97. 10.1016/j.ausmj.2020.04.001

[B4] Andrews A. M. Zhang N. Buechley C. Chapman R. Guillen J. L. Magee J. C.. (2016). Organ donation attitudes and practices among African Americans: an adapted measurement instrument. J. Health Care Poor Underserved 27, 1397–1410. 10.1353/hpu.2016.0118 27524775

[B5] Arisal I. Atalar T. (2020). Influence of knowledge, bodily integrity, religion, and media on attitudes toward organ donation on the university campus. Int. J. Nonprofit Voluntary Sector Market. 25:e1647. 10.1002/nvsm.1647

[B6] Aust F. Barth M. (2022). papaja: Prepare Reproducible APA Journal Articles with R Markdown. Available online at: https://github.com/crsh/papaja (Accessed April, 2025).

[B7] Bănescu V.. (2023). *Vasile Bănescu despre transplantul de organe: Biserica nu interzice nimic nimănui, ci doar invită la discernământ*. Available online at: https://basilica.ro/vasile-banescu-despre-transplantul-de-organe-biserica-nu-interzice-nimic-nimanui-ci-doar-invita-la-discernamant/ (Accessed April, 2025).

[B8] Babik I. Gardner E. S. (2021). Factors affecting the perception of disability: a developmental perspective. Front. Psychol. 12:702166. 10.3389/fpsyg.2021.702166 34234730 PMC8255380

[B9] Bacuşcă A. -E. Gavrilută C. Grecu S.-P. Tinica G. Ioan B. G. (2023). Evolution of attitudes of a Romanian urban population regarding organ donation. SAGE Open 13:21582440221147267. 10.1177/21582440221147267

[B10] Bacuşcă A. E. Burlacu A. Tinică G. Enache M. Tărus A. Gavrilută C.. (2022). Organ procurement, donation, and transplant awareness in an urban eastern European region: a general population survey. Ann. Transpl. 27:e938016. 10.12659/AOT.938016 36345227 PMC9653012

[B11] Bapat U. Kedlaya P. G. (2010). Organ donation, awareness, attitudes and beliefs among post graduate medical students. Saudi J. Kidney Dis. Transpl. 21, 174–180. 20061720

[B12] Barth M. (2022). tinylabels: Lightweight Variable Labels. Available online at: https://cran.r-project.org/package=tinylabels (Accessed April, 2025).

[B13] Bramstedt K. A. (2022). Arguments for ‘ocular donation' as standardised terminology to reduce the ‘Ick factor' of ‘eye donation'. J. Med. Ethics. 48, 935–936. 10.1136/medethics-2021-108003 34996863

[B14] Chukwuneke F. N. Ezenwugo A. C. (2022). Deontology vs. utilitarianism: understanding the basis for the moral theories in medicine. Int. J. Med. Health Dev. 27, 19–23. 10.4103/ijmh.IJMH_57_20

[B15] Clark N. L. Copping L. Swainston K. McGeechan G. J. (2023). Attitudes to organ donor registration in England under opt-out legislation. Progress Transpl. 33, 208–215. 10.1177/15269248231189869 37475461

[B16] Cohen E. L. Hoffner C. (2013). Gifts of giving: the role of empathy and perceived benefits to others and self in young adults' decisions to become organ donors. J. Health Psychol. 18, 128–138. 10.1177/1359105311433910 22322992

[B17] Cotrău P. Hodosan V. Vladu A. Daina L. Negrău M. Daina C.. (2020). Consent model, opt-in/opt-out system, and organ donation rates in European Union countries. Appl. Med. Inf. 42, 36–41.

[B18] Cotrău P. Negrău M. Hodoşan V. Vladu A. Daina C. M. Dulău D.. (2023). Organ donation awareness among family members of ICU patients. Medicina 59:11. 10.3390/medicina59111966 38004015 PMC10673166

[B19] Cronbach L. J. (1951). Coefficient alpha and the internal structure of tests. Psychometrika 16, 297–334. 10.1007/BF02310555

[B20] Da Silva I. R. F. Frontera J. A. (2015). Worldwide barriers to organ donation. JAMA Neurol. 72, 112–118. 10.1001/jamaneurol.2014.3083 25402335

[B21] Davis M. H. (1983a). Measuring individual differences in empathy: evidence for a multidimensional approach. J. Pers. Soc. Psychol. 44, 113–126. 10.1037//0022-3514.44.1.113

[B22] Davis M. H. (1983b). The effects of dispositional empathy on emotional reactions and helping: a multidimensional approach. J. Pers. 51, 167–184. 10.1111/j.1467-6494.1983.tb00860.x

[B23] de Groot J. van Hoek M. Hoedemaekers C. Hoitsma A. Smeets W. Vernooij-Dassen M.. (2015). Decision making on organ donation: the dilemmas of relatives of potential brain dead donors. BMC Med. Ethics 16:64. 10.1186/s12910-015-0057-1 26383919 PMC4574465

[B24] DiStefano C. Morgan G. B. (2014). A comparison of diagonal weighted least squares robust estimation techniques for ordinal data. Struct. Equ. Modeling 21, 425–438. 10.1080/10705511.2014.915373

[B25] Doerry K. Oh J. Vincent D. Fischer L. Schulz-Jürgensen S. (2022). Religious and cultural aspects of organ donation: narrowing the gap through understanding different religious beliefs. Pediatr. Transpl. 26:e14339. 10.1111/petr.14339 35735257

[B26] Evans J. Baronavski C. (2018). How do European Countries Differ in Religious Commitment? Use Our Interactive Map to Find Out. Available online at: https://policycommons.net/artifacts/617021/how-do-european-countries-differ-in-religious-commitment-use-our-interactive-map-to-find-out/1597768/ (accessed February 15, 2024).

[B27] Everett J. A. C. Faber N. S. Savulescu J. Crockett M. J. (2018). The costs of being consequentialist: social inference from instrumental harm and impartial beneficence. J. Exp. Soc. Psychol. 79, 200–216. 10.1016/j.jesp.2018.07.004 30393392 PMC6185873

[B28] Febrero B. Ríos A. López-Navas A. Martínez-Alarcón L. Almela-Baeza J. Sánchez J.. (2019). Psychological profile of teenagers toward organ donation: a multicentric study in Spain. Eur. J. Public Health 29, 1011–1018. 10.1093/eurpub/ckz036 30932155

[B29] Ferguson E. Murray C. O'Carroll R. E. (2019). Blood and organ donation: health impact, prevalence, correlates, and interventions. Psychol. Health 34, 1073–1104. 10.1080/08870446.2019.1603385 31213077

[B30] Ge F. Huang T. Yuan S. Zhou Y. Gong W. (2013). Gender issues in solid organ donation and transplantation. Ann. Transpl. 18, 508–514. 10.12659/AOT.889323 24064859

[B31] George P. Chittem M. Dwivedi R. Pal C. Guntupalli Y. Pati S.. (2022). The empathy factor: the role of empathy in knowledge, attitude, and practice of organ donation in India - a crossectional, observational study. Indian J. Transpl. 16:274. 10.4103/ijot.ijot_64_21

[B32] Georgiadou T. Fotakopoulou O. Pnevmatikos D. (2015). Adolescents' preferences for organ allocation: the role of empathy and altruism in allocation judgments. Eur. J. Dev. Psychol. 12, 310–323. 10.1080/17405629.2015.1010503

[B33] Ghoshal A. O'Carroll R. E. Ferguson E. Shepherd L. Doherty S. Mathew M.. (2022). Assessing medical mistrust in organ donation across countries using item response theory. J. Health Psychol. 27, 2806–2819. 10.1177/13591053211064985 34963351

[B34] Girlanda R. (2016). Deceased organ donation for transplantation: challenges and opportunities. World J. Transpl. 6, 451–459. 10.5500/wjt.v6.i3.451 27683626 PMC5036117

[B35] Grigoras I. Condac C. Cartes C. Blaj M. Florin G. (2010). Presumed consent for organ donation: is Romania prepared for it? Transpl. Proc. 42, 144–146. 10.1016/j.transproceed.2009.12.006 20172301

[B36] Guedj M. Sastre M. T. M. Mullet E. (2011). Donating organs: a theory-driven inventory of motives. Psychol. Health Med. 16, 418–429. 10.1080/13548506.2011.555770 21749239

[B37] Hansen S. L. Eisner M. I. Pfaller L. Schicktanz S. (2018). “Are you in or are you out?!” Moral appeals to the public in organ donation poster campaigns: a multimodal and ethical analysis. Health Commun. 33, 1020–1034. 10.1080/10410236.2017.1331187 28622010

[B38] Holman A. Beatrice I. (2016). The malfunctions of a national transplantation system: multi-layered explanations from within. Revista Medico-Chirurgicala a Societatii De Medici Si Naturalisti Din Iasi 120, 62–69. 27125074

[B39] Holman A. Karner-Huţuleac A. (2017). “Press portrayals of the psychological experiences of people involved in organ transplantation,” in Recent Trends in Social Systems: Quantitative Theories and Quantitative Models, eds. A. Maturo, Š. Hošková-Mayerová, D.-T. Soitu, and J. Kacprzyk (Springer International Publishing), 281–293. 10.1007/978-3-319-40585-8_25

[B40] Holman A. Karner-Hutuleac A. Ioan B. (2013). Factors of the willingness to consent to the donation of a deceased family member's organs among the Romanian urban population. Transpl. Proc. 45, 3178–3182. 10.1016/j.transproceed.2013.05.009 24182780

[B41] Hyde M. K. White K. M. (2009). To be a donor or not to be? Applying an extended theory of planned behavior to predict posthumous organ donation intentions. J. Appl. Soc. Psychol. 39, 880–900. 10.1111/j.1559-1816.2009.00464.x

[B42] Kahane G. (2015). Sidetracked by trolleys: why sacrificial moral dilemmas tell us little (or nothing) about utilitarian judgment. Soc. Neurosci. 10, 551–560. 10.1080/17470919.2015.1023400 25791902 PMC4642180

[B43] Kahane G. Everett J. A. C. Earp B. D. Caviola L. Faber N. S. Crockett M. J.. (2018). Beyond sacrificial harm: a two-dimensional model of utilitarian psychology. Psychol. Rev. 125, 131–164. 10.1037/rev0000093 29265854 PMC5900580

[B44] Khoshravesh S. Karimi-Shahanjarini A. Poorolajal J. Bashirian S. Barati M. Hamidi M. (2019). Socio-cultural Factors Contributing to Being an Organ Donor in a Religious Context. Available online at: https://www.researchsquare.com/article/rs-2016/latest (Accessed April, 2025). 10.1177/0272684X20972836 33241985

[B45] Kong S. Lee Y.-H. (2021). How perceived similarity moderates sympathy and pride appeal in organ donation messages. Transpl. Proc. 53, 2105–2111. 10.1016/j.transproceed.2021.07.010 34420779

[B46] Krupic F. Westin O. Hagelberg M. Sköldenberg O. Samuelsson K. (2019). The influence of age, gender and religion on willingness to be an organ donor: experience of religious muslims living in Sweden. J. Relig. Health 58, 847–859. 10.1007/s10943-018-0670-7 30006834 PMC6522646

[B47] Lay S. Zagefka H. González R. Álvarez B. Valdenegro D. (2020). Don't forget the group! The importance of social norms and empathy for shaping donation behaviour. Int. J. Psychol. 55, 518–531. 10.1002/ijop.12626 31608442

[B48] Li M. T. Hillyer G. C. Husain S. A. Mohan S. (2019). Cultural barriers to organ donation among Chinese and Korean individuals in the United States: a systematic review. Transpl. Int. 32, 1001–1018. 10.1111/tri.13439 30968472 PMC6867085

[B49] López J. S. Soria-Oliver M. Aramayona B. García-Sánchez R. Martínez J. M. Martín M. J. (2018). An integrated psychosocial model of relatives' decision about deceased organ donation (IMROD): joining pieces of the puzzle. Front. Psychol. 9:408. 10.3389/fpsyg.2018.00408 29692744 PMC5902731

[B50] Maftei A. Gherguţ A. Roca D. Dănilă O. (2023). Transitioning from decades of segregation: religiosity and the attitudes towards intellectual disability in romania. J. Beliefs Values 44, 334–348. 10.1080/13617672.2022.2125674

[B51] Manstead A. S. R. (2000). “The role of moral norm in the attitude–behavior relation,” in Attitudes, Behavior, and Social Context: The Role of Norms and Group Membership (Lawrence Erlbaum Associates Publishers), 11–30. Available online at: https://psycnet.apa.org/record/1999-04360-001 (Accessed April, 2025).

[B52] Mardia K. V. (1970). Measures of multivariate skewness and kurtosis with applications. Biometrika 57, 519–530. 10.1093/biomet/57.3.519

[B53] Mekkodathil A. El-Menyar A. Sathian B. Singh R. Al-Thani H. (2020). Knowledge and willingness for organ donation in the middle eastern region: a meta-analysis. J. Relig. Health 59, 1810–1823. 10.1007/s10943-019-00883-x 31309441 PMC7359145

[B54] Miller J. Currie S. O'Carroll R. E. (2019). ‘What if I'm not dead?' – Myth-busting and organ donation. Br. J. Health Psychol. 24, 141–158. 10.1111/bjhp.12344 30345605 PMC6587533

[B55] Mocan N. Tekin E. (2007). The determinants of the willingness to donate an organ among young adults: evidence from the United States and the European Union. Soc. Sci. Med. 65, 2527–2538. 10.1016/j.socscimed.2007.07.004 17765372

[B56] Morgan S. E. Harrison T. R. Afifi W. A. Long S. D. Stephenson M. T. (2008a). In their own words: the reasons why people will (not) sign an organ donor card. Health Commun. 23, 23–33. 10.1080/10410230701805158 18443990

[B57] Morgan S. E. Stephenson M. T. Harrison T. R. Afifi W. A. Long S. D. (2008b). Facts versus ‘feelings': how rational is the decision to become an organ donor? J. Health Psychol. 13, 644–658. 10.1177/1359105308090936 18519438

[B58] Murphy T. F. Veatch R. M. (2006). Members first: the ethics of donating organs and tissues to groups. Cambr. Q. Healthc. Ethics 15, 50–59. 10.1017/S0963180106060063 16529307

[B59] O'Carroll R. E. Foster C. McGeechan G. Sandford K. Ferguson E. (2011). The “Ick” factor, anticipated regret, and willingness to become an organ donor. Health Psychol. 30, 236–245. 10.1037/a0022379 21401258

[B60] Petre O. A. Băban A. (2022). Organ donation in Romanian online media: a content analysis. Euro. J. Health Commun. 3:3. 10.47368/ejhc.2022.302

[B61] Pfaller L. Hansen S. L. Adloff F. SchIcktanz S. (2018). ‘Saying no to organ donation': an empirical typology of reluctance and rejection. Sociol. Health Illness 40, 1327–1346. 10.1111/1467-9566.12775 29956337

[B62] QuIck B. L. Reynolds-Tylus T. Fico A. E. Feeley T. H. (2016). An investigation into mature adults' attitudinal reluctance to register as organ donors. Clin. Transpl. 30, 1250–1257. 10.1111/ctr.12815 27459632

[B63] R Core Team R. R. (2022). Foreign: Read Data Stored by'minitab','s','SAS','SPSS','stata','systat','weka','dBase',... Available online at: https://CRAN.R-project.org/package=foreign (Accessed April, 2025).

[B64] R Core Team R. R. (2023). R: A Language and Environment for Statistical Computing. R Foundation for Statistical Computing. Available online at: https://www.R-project.org/ (Accessed April, 2025).

[B65] Randhawa G. Neuberger J. (2016). Role of religion in organ donation-development of the United Kingdom faith and organ donation action plan. Transpl. Proc. 48, 689–694. 10.1016/j.transproceed.2015.10.074 27234715

[B66] Riess H. (2017). The science of empathy. J. Patient Exp. 4, 74–77. 10.1177/2374373517699267 28725865 PMC5513638

[B67] Rodrigue J. R. Cornell D. L. Jackson S. I. Kanasky W. Marhefka S. Reed A. I. (2004). Are organ donation attitudes and beliefs, empathy, and life orientation related to donor registration status?. Progress Transpl. 14, 56–60. 10.1177/152692480401400109 15077739

[B68] Romanian Patriarchate (2009). Organ Transplantation. Archived from Patriarhia.ro. Available online at: https://arhiva.patriarhia.ro/transplantul-de-organe-1451.html (Retrieved April, 2025).

[B69] Ruggieri S. Boca S. Ingoglia S. (2023). Willingness to donate organs after death. Euro. J. Health Psychol. 30, 51–64. 10.1027/2512-8442/a000118

[B70] Russell E. Robinson D. H. Z. Thompson N. J. Perryman J. P. Arriola K. R. J. (2012). Distrust in the healthcare system and organ donation intentions among African Americans. J. Commun. Health 37, 40–47. 10.1007/s10900-011-9413-3 21626439 PMC3489022

[B71] Samoila R. (2010). Why We Don't Want to Donate Organs. Available online at: https://health.ec.europa.eu/system/files/2016-11/art_romania_2010_en_0.pdf (Accessed April, 2025).

[B72] Satorra A. (2000). “Scaled and adjusted restricted tests in multi-sample analysis of moment structures,” in Innovations in Multivariate Statistical Analysis: A Festschrift for Heinz Neudecker, eds. R. D. H. Heijmans, D. S. G. Pollock, and A. Satorra (Springer US), 233–247. 10.1007/978-1-4615-4603-0_17

[B73] Scholz N. (2020). Organ Donation and Transplantation: Facts, Figures and European Union Action. Available online at: https://policycommons.net/artifacts/1337208/organ-donation-and-transplantation/1944888/ (Accessed April, 2025).

[B74] Shah M. B. Vilchez V. Goble A. Daily M. F. Berger J. C. Gedaly R.. (2018). Socioeconomic factors as predictors of organ donation. J. Surg. Res. 221, 88–94. 10.1016/j.jss.2017.08.020 29229159

[B75] Shin Y. Kim S. Kim D. H. Lee S. Cho M. Ihm J. (2022). The effect of deliberative process on the self-sacrificial decisions of utilitarian healthcare students. BMC Med. Ethics 23:28. 10.1186/s12910-022-00769-w 35305638 PMC8933755

[B76] Skowronski J. J. (1997). On the psychology of organ donation: attitudinal and situational factors related to the willingness to be an organ donor. Basic Appl. Soc. Psych. 19, 427–456. 10.1207/s15324834basp1904_3

[B77] Statter M. B. Noritz G. Macauley R. C. Geis G. M. Laventhal N. T. Opel D. J.. (2020). Children with intellectual and developmental disabilities as organ transplantation recipients. Pediatrics 145:e20200625. 10.1542/peds.2020-0625 32312907

[B78] Tesema B. Bogale E. K. Wasihun Y. Anagaw T. F. (2023). Intention to donate kidney and associated factors among students in bahir dar university: application of theory of planned behavior. Int. J. General Med. 16, 5363–5376. 10.2147/IJGM.S441636 38021069 PMC10674569

[B79] Thornton J. D. Wong K. A. Cardenas V. Curtis J. R. Spigner C. Allen M. D. (2006). Ethnic and gender differences in willingness among high school students to donate organs. J. Adolescent Health 39, 266–274. 10.1016/j.jadohealth.2005.12.028 16857540

[B80] Todeancă D. Holman A. Turliuc M. Antonovici L. (2019). The social representation of organ donation in the Romanian online environment. A qualitative approach. Psihologia Sociala 43:93.

[B81] Tumin M. Tafran K. Tang L. Y. Chong M. C. Mohd Jaafar N. I. Mohd Satar N.. (2016). Factors associated with medical and nursing students' willingness to donate organs. Medicine 95:e3178. 10.1097/MD.0000000000003178 27015207 PMC4998402

[B82] Umair S. Ho J.-A. Ng S. S. I. Basha N. K. (2023). Moderating role of religiosity and the determinants to attitude, willingness to donate and willingness to communicate posthumous organ donation decisions among university students in Pakistan. OMEGA J. Death Dying 88, 216–244. 10.1177/00302228211045170 34505539

[B83] Viens A. M. (2016). “Bodily integrity as a barrier to organ donation,” in Organ Transplantation in Times of Donor Shortage: Challenges and Solutions, eds. R. J. Jox, G. Assadi, and G. Marckmann (Springer International Publishing), 19–26. 10.1007/978-3-319-16441-0_3

[B84] Volk M. L. Lok A. S. Ubel P. A. Vijan S. (2008). Beyond utilitarianism: a method for analyzing competing ethical principles in a decision analysis of liver transplantation. Med. Decis. Mak. 28, 763–772. 10.1177/0272989X08316999 18725405

[B85] Wei C. Yu Z. Li Y. (2021). Empathy impairs virtue: the influence of empathy and vulnerability on charitable giving. Internet Res. 31, 1803–1822. 10.1108/INTR-07-2020-0407

[B86] Wilczek-Rużyczka E. Milaniak I. Przybyłowski P. WierzbIcki K. Sadowski J. (2014). Influence of empathy, beliefs, attitudes, and demographic variables on willingness to donate organs. Transpl. Proc. 46, 2505–2508. 10.1016/j.transproceed.2014.09.024 25380854

[B87] William R. (2023). Psych: Procedures for Psychological, Psychometric, and Personality Research. Northwestern University. Available online at: https://CRAN.R-project.org/package=psych (Accessed April, 2025).

[B88] Williamson L. D. Bigman C. A. QuIck B. L. (2019). A qualitative examination of African Americans' organ donation-related medical mistrust beliefs. Howard J. Commun. 30, 430–445. 10.1080/10646175.2018.1512064

[B89] Zhu H. (2021). kableExtra: Construct Complex Table with ‘Kable' and Pipe Syntax. Available online at: https://CRAN.R-project.org/package=kableExtra (Accessed April, 2025).

